# Glucocorticoid activates STAT3 and NF-κB synergistically with inflammatory cytokines to enhance the anti-inflammatory factor TSG6 expression in mesenchymal stem/stromal cells

**DOI:** 10.1038/s41419-024-06430-1

**Published:** 2024-01-18

**Authors:** Peiqing Huang, Rongrong Sun, Chenchang Xu, Zixuan Jiang, Muqiu Zuo, Yinghong Li, Rui Liu, Pixia Gong, Yuyi Han, Jiankai Fang, Peishan Li, Changshun Shao, Yufang Shi

**Affiliations:** The First Affiliated Hospital of Soochow University, State Key Laboratory of Radiation Medicine and Protection, Institutes for Translational Medicine, Soochow University Suzhou Medical College, Suzhou, China

**Keywords:** Mesenchymal stem cells, Immunotherapy

## Abstract

Glucocorticoid (GC) is essential for maintaining immune homeostasis. While GC is known to regulate the expression of genes related to inflammation in immune cells, the effects of GC, especially in the presence of inflammation, on non-immune cells remain largely unexplored. In particular, the impact of GC on inflammatory cytokine-induced immune modulatory responses of tissue stromal cells is unknown, though it has been widely used to modulate tissue injuries. Here we found that GC could enhance the expression of TSG6, a vital tissue repair effector molecule, in IFNγ and TNFα treated human umbilical cord (UC)-MSCs. NF-κB activation was found to be required for GC-augmented TSG6 upregulation. STAT3, but not STAT1, was also found to be required for the TSG6 upregulation in MSCs exposed to IFNγ, TNFα and GC. Moreover, the phosphorylation (activation) of STAT3 was attenuated when NF-κB was knocked down. Importantly, human UC-MSCs pretreated with a cocktail containing GC, IFNγ, and TNFα could significantly enhance the therapeutic effect of human UC-MSCs in an acute lung injury mouse model, as reflected by reduced infiltration of immune cells and down-regulation of iNOS in macrophages in the lung. Together, the findings reveal a novel link between GR, NF-κB and STAT3 in regulating the immunomodulatory and regenerative properties of MSCs, providing novel information for the understanding and treatment of inflammatory conditions.

## Introduction

Glucocorticoid (GC) is a class of steroid hormones. Endogenous glucocorticoid production is induced upon the hypothalamic-pituitary-adrenal axis (HPA axis) activation, especially under stress conditions. The binding of GC to the glucocorticoid receptor (GR) regulates the expression of many genes. GC has been widely used to treat various immune-related diseases. At the same time, GC achieved exciting therapeutic effects in treating tissue injuries, like acute spinal cord injury [1, 2] and acute lung injury [3], which were related to the induction of anti-inflammatory and tissue repair effector molecules [4]. In the battle combating COVID-19, the American College of Rheumatology treatment guideline recommends intravenous high-dose GC as a first-line therapy in multisystem inflammatory syndromes [5].

For decades, most of studies of GC have been focused on immune cells. While it is possible that GC may also act on other cell types to exert its immunomodulatory effect, the effects of GC on non-immune cells, like tissue stromal cells, remain largely unexplored. Mesenchymal stem/stromal cells (MSCs) reside in almost all tissues and have attracted much attention for their immunoregulation capability and enhancement of tissue repair in mouse models of acute lung injury [6], myocardial infarction [7], corneal injury [8], peritonitis [9], and arthritis [10]. MSCs are known to exert their immunoregulatory and reparative functions via producing an array of effector molecules, including tumor necrosis factor (TNF)-stimulated gene 6 protein (TSG6), a 30-kDa hyaluronan-binding protein. In-vitro-expanded MSCs used for therapeutic treatment of acute lung injury [11] and inflammatory bowel disease (IBD) [12, 13] have achieved excellent efficacy through expressing TSG6. A cocktail of proinflammatory cytokines, IFNγ concomitant with TNFα, was shown to have the capability to enable MSCs with the ability to suppress immune responses and to increase the reparative function [14], through promoting the secretion of several factors, especially TSG6, and metabolites [15]. However, how TSG6 expression is regulated and how it functions in inflammatory settings have not been fully elucidated. The binding of steroids to GR was reported to inhibit the dissociation of IκB from NF-κB to restrain the expression of pro-inflammatory genes downstream of NF-κB [16, 17]. Here we showed that GC could boost the expression of TSG6 in the presence of both IFNγ and TNFα. We further demonstrated that GC could increase the phosphorylation of STAT3, the main downstream transcription factor of IFNγ signaling. Meanwhile, GC treatment could further enhance the therapeutic capacity of IFNγ and TNFα-stimulated human MSCs in lipopolysaccharide (LPS)-induced acute lung injury (ALI). Therefore, our study reveals a novel link between GR, NF-κB and STAT3 in the regulation of TSG6 in MSCs and has implications in developing a new strategy that will optimize MSCs-based clinical treatments for tissue injury conditions.

## Results

### GC promotes the expression of TSG6 in IFNγ and TNFα-activated human MSCs

**Fig. 1:**
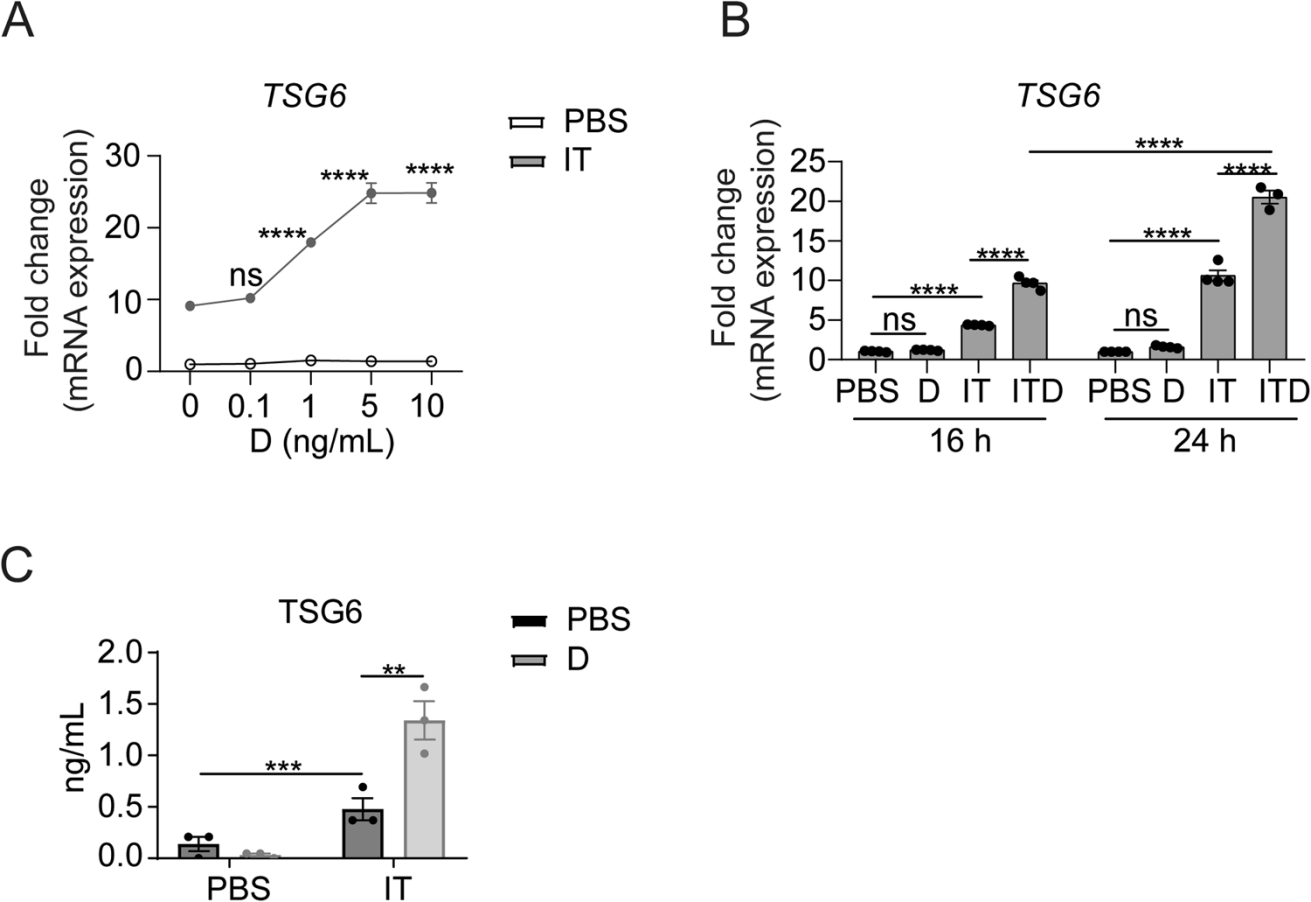
The expression of TSG6 is significantly increased by dexamethasone in IFNγ and TNFα-activated MSCs. **A** mRNA levels of *TSG6* in MSCs treated with PBS, IT (10 ng/mL each) combined with different doses of dexamethasone (D) (0, 0.1, 1, 5, 10 ng/mL) were determined by qRT-PCR 24 h after treatment (*n* = 3 or 4). **B** mRNA levels of *TSG6* in MSCs under PBS, D, IT or ITD (10 ng/mL each) stimulation for 16 h or 24 h were determined by qRT-PCR (*n* = 3 or 4). **C** Protein levels of TSG6 in MSCs culture supernatant under PBS, D, IT or ITD (10 ng/mL each) stimulation for 24 h were determined by ELISA (*n* = 3).

TSG6 was mainly expressed in human MSCs upon stimulation by inflammatory factors, especially the combination of IFNγ and TNFα [11]. We set out to explore the effects of GC on TSG6 expression in MSCs. We added recombinant human TNFα and IFNγ in combination with dexamethasone, which we refer to as ITD, to cultured MSCs. Consistent with previous studies, TNFα and IFNγ treatment, referred to as IT, induced the expression of TSG6 (Fig. 1A, B, C). Surprisingly, while dexamethasone alone did not induce TSG6, it substantially further up-regulated the expression of TSG6 induced by IFNγ and TNFα (Fig. 1A, B, C). The augmenting effect of dexamethasone on TSG6 expression reached the plateau at 5 ng/mL. Thus, GC could act synergistically with IFNγ and TNFα to upregulate the expression of a critical immune modulatory and regenerative molecule, TSG6, in MSCs.

### GC promotes TSG6 expression via GR

**Fig. 2:**
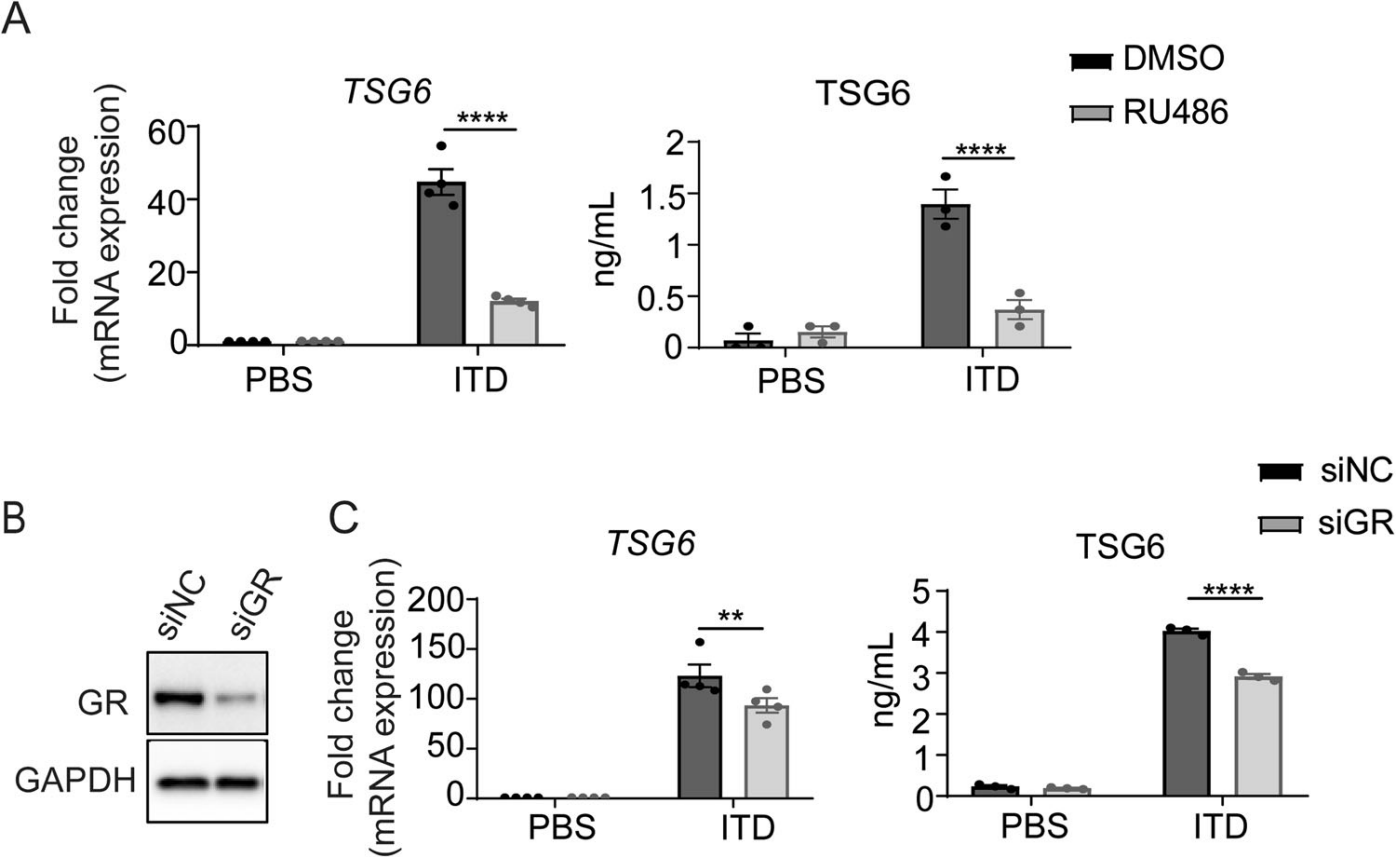
Dexamethasone promotes TSG6 expression through GR. **A** mRNA and protein levels of TSG6 in MSCs under PBS or ITD (10 ng/mL each) in a combination with DMSO or RU486 (2 μM) stimulation for 24 h were determined by qRT-PCR or ELISA (*n* = 3 or 4). **B** Protein level of GR in GR-knockdown MSCs was measured by Western blotting analysis. (*n* = 3) **C** mRNA and protein levels of TSG6 in NC-knockdown MSCs or GR-knockdown MSCs under PBS or ITD (10 ng/mL each) stimulation for 24 h were determined by qRT-PCR or ELISA (*n* = 3 or 4).

Although GC mainly acts through GR, it has been reported that GC also functions in a GR-independent manner [18–23]. We set to test whether the positive regulatory effect of dexamethasone on TSG6 expression is mediated by GR. RU486, a widely used competitive inhibitor of GC, was employed to block the activation of GR by GC. The mRNA expression and protein levels of TSG6 stimulated by ITD were both dramatically downregulated when RU486 was present (Fig. 2A). We next knocked down the expression of GR in MSCs by small interfering RNA (siRNA). The knockdown efficiency was verified by the reduction in GR protein level and downregulation of a known GR transcriptional target gene, *HSD11B1* [24, 25]. (Fig. 2B, Fig. S1A). And we found that in GR-knockdown MSCs, the upregulation of TSG6 expression enabled by dexamethasone was much weakened (Fig. 2C). These results strongly suggest that the upregulation of TSG6 by ITD stimulation was achieved through GR.

### GC promotes the expression of TSG6 only in the presence of both IFNγ and TNFα

**Fig. 3:**
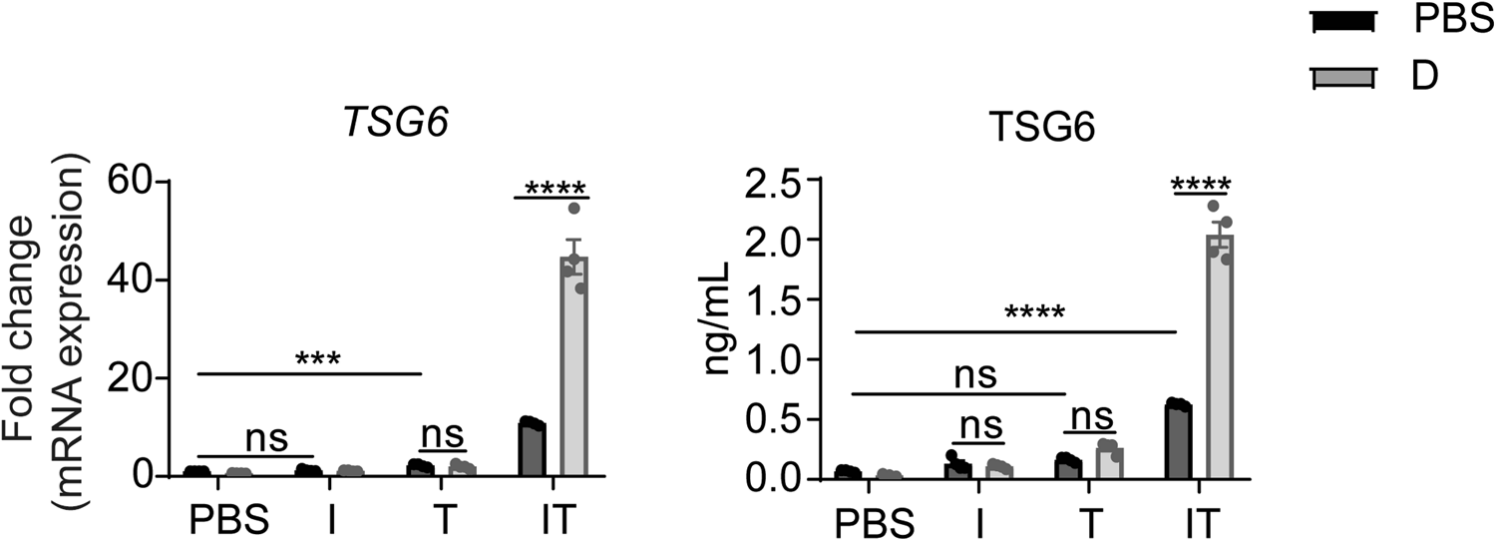
Dexamethasone promotes TSG6 expression synergistically with TNFα and IFNγ . mRNA and protein levels of TSG6 in MSCs under PBS, IFNγ (I), TNFα (T) or IT (10 ng/mL each) in a combination with PBS or dexamethasone (D) (10 ng/mL) stimulation for 24 h were determined by qRT-PCR or ELISA (*n* = 3 or 4).

To test whether dexamethasone could promote TSG6 expression synergistically with IFNγ or TNFα, we treated MSCs with dexamethasone only, plus IFNγ or plus TNFα. Interestingly, neither combination could further promote TSG6 expression. Dexamethasone could only further upregulate TSG6 in the presence of both IFNγ and TNFα (Fig. 3), which means that for dexamethasone to upregulate TSG6, GC, IFNγ and TNFα downstream signals need to act together.

### NF-κB mediates the regulatory effect of GC on TSG6 expression

**Fig. 4:**
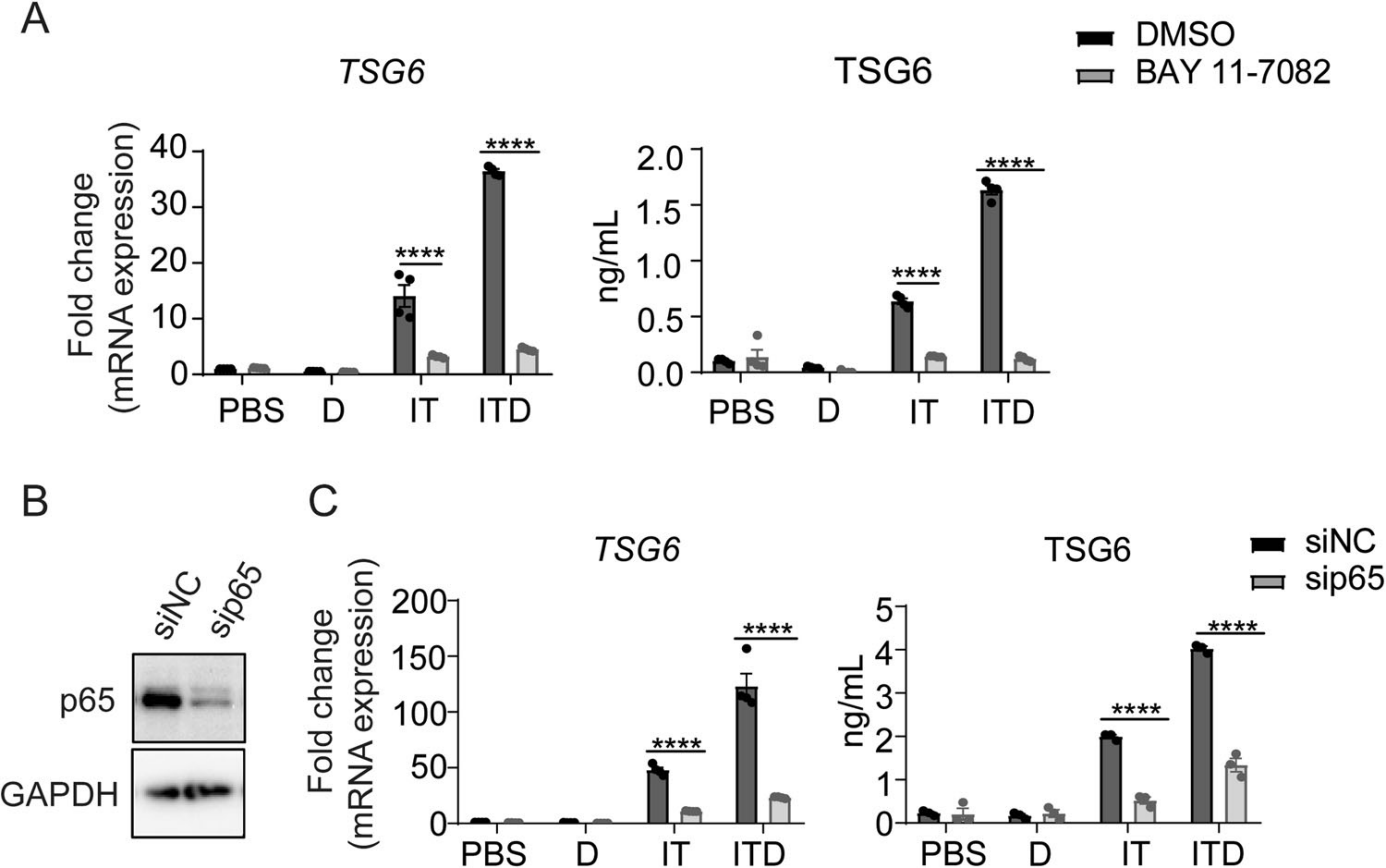
NF-κB mediates the upregulation of TSG6 by dexamethasone. **A** mRNA and protein levels of TSG6 in MSCs stimulated by PBS, D, IT or ITD (10 ng/mL each) in a combination with DMSO or BAY 11-7082 (2 μM) for 24 h were determined by qRT-PCR or ELISA (*n* = 3 or 4). **B** Protein level of p65 in p65-knockdown MSCs was measured by Western blotting analysis. (*n* = 3) **C** mRNA and protein levels of TSG6 in NC-knockdown MSCs or p65-knockdown MSCs under PBS, D, IT or ITD (10 ng/mL each) stimulation for 24 h were determined by qRT-PCR or ELISA (*n* = 3 or 4).

Since TNFα mainly acts through NF-κB signaling, we determined the TSG6 expression in the presence of NF-κB inhibitor BAY 11-7082. We observed that the inhibitor could significantly suppress the expression of TSG6 and abrogate the expression-promoting effect of dexamethasone on TSG6 (Fig. 4A). Then we knocked down p65, an NF-κB subunit, through siRNA, to verify the role of NF-κB signaling in the regulatory effect of GC on TSG6 (Fig. 4B). As expected, dexamethasone treatment could no longer up-regulate TSG6 under ITD stimulation in p65-knockdown MSCs (Fig. 4C). These results indicated that NF-κB mediates the regulatory effect of GC on TSG6 expression in IFNγ and TNFα-activated MSCs.

### STAT3 and NF-κB synergistically mediate TSG6 upregulation

**Fig. 5:**
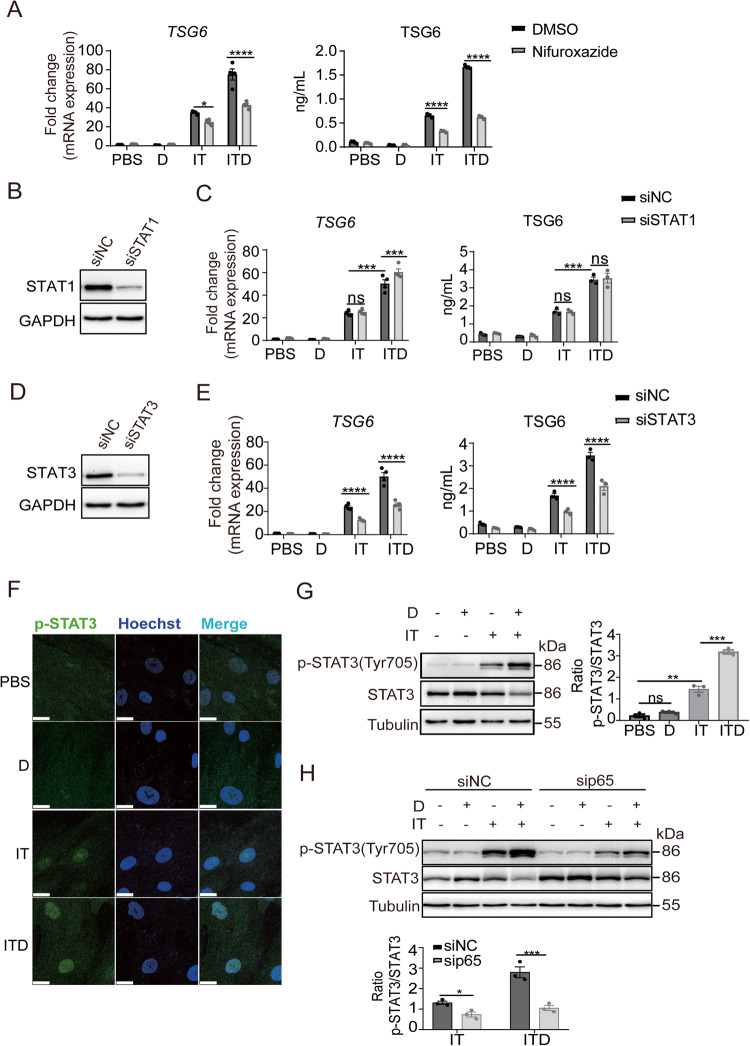
STAT3 is required for the upregulation of TSG6 by GC. **A** mRNA and protein levels of TSG6 in MSCs treated with PBS, D, IT or ITD (10 ng/mL each) with or without DMSO or nifuroxazide (40 μM) were determined by qRT-PCR or ELISA after 24 h (*n* = 3 or 4). **B** Protein level of STAT1 in STAT1-knockdown MSCs was measured by Western blotting analysis (*n* = 3). **C** mRNA and protein levels of TSG6 in NC-knockdown MSCs or STAT1-knockdown MSCs under PBS, D, IT or ITD (10 ng/mL each) stimulation for 24 h were determined by qRT-PCR or ELISA (*n* = 3 or 4). **D** Protein level of STAT3 in STAT3-knockdown MSCs was measured by Western blotting analysis (*n* = 3). **E** mRNA and protein levels of TSG6 in NC-knockdown MSCs or STAT3-knockdown MSCs under PBS, D, IT or ITD (10 ng/mL each) stimulation for 24 h were determined by qRT-PCR or ELISA (*n* = 3 or 4). **F** Phospho-STAT3 (Tyr705) in MSCs stimulated by PBS, D, IT or ITD (10 ng/mL each) for 1 h was localized by immunofluorescence staining. Scar bar = 23.2 μm (*n* = 3). **G** Protein levels of p-STAT3 (Tyr705), STAT3 and Tubulin in MSCs under PBS, D, IT or ITD (10 ng/mL each) stimulation for 24 h were determined by Western blotting analysis (*n* = 3 or 4). **H** Protein levels of p-STAT3 (Tyr705), STAT3 and Tubulin in NC-knockdown MSCs or p65-knockdown MSCs under PBS, D, IT or ITD (10 ng/mL each) stimulation for 24 h were determined by Western blotting analysis (*n* = 3).

IFNγ signals commonly via the JAK-STAT pathways [26]. We applied nifuroxazide, a JAK-STAT inhibitor, to MSCs in combination with IT or ITD stimulation and found that the expression of TSG6 in MSCs was downregulated by nifuroxazide under IT or ITD stimulation. At the same time, the enhancement of TSG6 expression by GC was suppressed (Fig. 5A). These results showed that the JAK-STAT pathways were indeed involved in TSG6 expression. In the STAT family, STAT1 is usually regarded as the primary mediator of IFNγ effects. Thus, we knocked down STAT1 by siRNA in MSCs, and checked the expression of TSG6. The knockdown efficiency was measured by STAT1 protein level and mRNA level of a known target gene, *ICAM1* [27] (Fig. 5B, S1B). To our surprise, in STAT1-knockdown MSCs, TSG6 expression remained unchanged under IT or ITD stimulation (Fig. 5C). Considering that STAT3 could mediate the inhibitory effect of nifuroxazide on the JAK-STAT pathways [28, 29], we knocked down STAT3 in MSCs, and subjected the cells to the same treatment as for STAT1-knockdown MSCs and found that the expression of TSG6 was decreased by STAT3 knockdown in both IT group and ITD group(Fig. 5E). The knockdown efficiency was determined by the STAT3 protein level and mRNA level of a known target gene, *ICAM1* [30] (Fig. 5D, S1B). STAT3 activation requires phosphorylation of a critical tyrosine residue (Tyr705), which mediates its dimerization and is a prerequisite for nucleus entry and DNA binding [31]. Therefore, we examined the phosphorylation of STAT3 in MSCs. We observed nuclear translocation of p-STAT3 (Tyr705) in both IT group and ITD group (Fig. 5F), strongly suggesting that STAT3 was activated. Interestingly, ITD stimulation could induce the phosphorylation of the STAT3 tyrosine residue (Tyr705) to a greater extent than IT stimulation (Fig. 5G). We then wondered if this enhanced STAT3 phosphorylation was mediated by NF-κB. Thus, we examined STAT3 phosphorylation levels in MSCs in which p65 was knocked down by siRNA. After p65 knockdown, the phosphorylation level of STAT3 was significantly decreased when MSCs were stimulated by IT or ITD, and the enhanced STAT3 phosphorylation by dexamethasone was eliminated (Fig. 5H). The results strongly suggested that STAT3, rather than STAT1, was involved in TSG6 regulation under IT or ITD stimulation in MSCs.

### ITD-pretreated MSCs show stronger anti-inflammatory effects than IT-pretreated MSCs

**Fig. 6:**
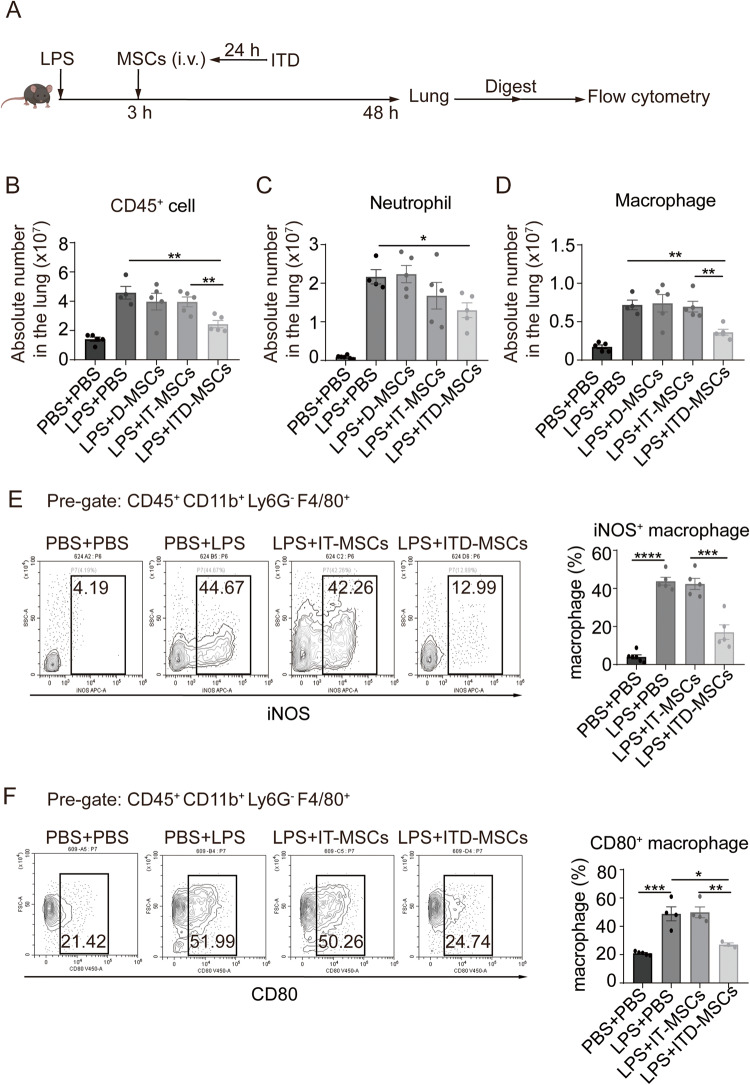
ITD-MSCs exhibit stronger anti-inflammatory effect than IT-MSCs. **A** Model diagram of acute lung injury treatment. **B**, **C**, **D** Absolute number of immune cell (CD45^+^ cell), neutrophil (CD45^+^ CD11b^+^ Ly6G^+^ cell), macrophage (CD45^+^ CD11b^+^ Ly6G^−^ F4/80^+^ cell) in the lung of mouse treated with PBS + PBS, LPS + PBS, LPS + D-MSCs, LPS + IT-MSCs or LPS + ITD-MSCs was counted by flow cytometry at 48 h after LPS infusion (*n* = 3, 4 or 5). **E**, **F** Percent of iNOS^+^ cells or CD80^+^ cells in macrophages in the lungs of ALI mice treated with PBS + PBS, LPS + PBS, LPS + IT-MSCs or LPS + ITD-MSCs were counted by flow cytometry at 48 h after LPS infusion (*n* = 3, 4 or 5).

In-vitro expanded IT-pretreated MSCs have been reported to have a potent anti-inflammatory effect in the treatment of acute lung injury and inflammatory bowel disease animal models, which was mainly mediated by the expression of TSG6 [11–13]. Would the ITD-pretreated MSCs (ITD-MSCs) exhibit a more potent anti-inflammatory effect than IT-pretreated MSCs (IT-MSCs)? To address this, we applied ITD-MSCs and IT-MSCs, respectively, to the LPS-induced acute lung injury (ALI) mouse model (Fig. 6A). To accentuate the difference in efficacy between the IT-MSCs and ITD-MSCs, we chose to give a smaller dosage of MSCs infusion, in which ITD-MSCs were shown to inhibit the aggregation of immune cells while IT-MSCs had no therapeutic effect (Fig. 6B). As the main infiltrating immune cells at the site of inflammation are neutrophils and macrophages in the ALI mouse model, we measured the number of the two types of immune cells in the injured lung. ITD-MSCs could decrease the accumulation of neutrophils and macrophages in the case of small MSCs number infusion, while IT-MSCs did not (Fig. 6C, D). We also used dexamethasone-pretreated MSCs (D-MSCs) to treat ALI mouse model in order to exclude the dexamethasone residue after pre-treatment of MSCs in vitro. The number of immune cells, neutrophils and macrophages in the injured lung was not reduced by D-MSCs, indicating that the anti-inflammatory effect of ITD-MSCs was not due to dexamethasone residue in MSCs (Fig. 6B, C, D). iNOS has been used to define classically activated M1, the pro-inflammatory phenotype [32]. We noticed that ITD-MSCs reduced the expression of iNOS in the accumulated macrophages in the injured lung, while IT-MSCs did not (Fig. 6E). Furthermore, a similar phenomenon was observed in type I macrophage polarization after ITD-MSCs or IT-MSCs injection in the injured lung (Fig. 6F). These results indicated that ITD-MSCs could reduce the accumulation of inflammatory cells and inhibit the pro-inflammatory capability of macrophages more strongly than IT-MSCs.

## Discussion

Steroid medications, such as dexamethasone, are the drugs of first choice for inflammation, pain, as well as anemia. In patients suffering from severe wound, the application of steroids is believed to reduce inflammation and pain. However, the direct effects of steroids on tissue regeneration are rarely considered. We showed in this study that dexamethasone could further enhance the expression of TSG6, a classical anti-inflammatory and tissue regeneration factor, in the presence of IFNγ and TNFα. This suggests that in the inflammatory microenvironment of injured tissues, injury-induced GC may regulate the repair of damaged tissues by regulating the function of stromal cells through synergistic action with inflammatory factors. Further, by pretreating the in-vitro*-*expanded MSCs with GC and inflammatory cytokines to treat ALI mice, we found that ITD-pretreated MSCs could more effectively reduce the number of infiltrating macrophages but not neutrophils in the lung of ALI mice than IT-pretreated MSCs. Meanwhile, ITD-pretreated MSCs were more effective in inhibiting the proinflammatory activity of macrophages than IT-pretreated MSCs. Thus, in addition to directly acting on immune cells, GC may also increase the immunoregulatory function of MSCs to promote the resolution of inflammation and regeneration. Considering that TSG6 is responsible for inhibiting monocyte/macrophage recruitment [33], and for converting macrophages from a proinflammatory to an anti-inflammatory phenotype in ALI therapy [34], TSG6 may have presumably mediated the increased therapeutic effect of ITD-pretreated MSCs over IT-pretreated MSCs.

TNFα commonly activates the NF-κB signaling pathway. Consistently, we found that NF-κB mediated the expression of TSG6 under IT or ITD stimulation. While GR and NF-κB generally display mutually antagonistic effects, genomic analysis revealed that certain combinations of stimuli can cause GR and NF-κB to exert synergistic effects on a subset of genes [35, 36]. Furthermore, cryptic GR-binding sites pervading genomic NF-κB response elements were identified [37]. And our results of TSG6 expression under ITD stimuli also indicate the synergistic effect of GR and NF-κB.

IFNγ usually functions via the JAK-STAT1 signaling, though there are alternate pathways [38]. We demonstrated that STAT3, but not STAT1, mediates TSG6 expression under both IT and ITD stimulation conditions. It was reported that IFNs signatures may greatly overlap and are difficult to distinguish as they can also activate other STAT homodimers in a context-dependent manner [39, 40]. The signal transduction process of STAT family under IT or ITD stimulation needs further investigation. STAT3 was reported to cooperate with GR or p65 to drive gene expression in tumor cells and T cells [41–44]. Interestingly, we observed the synergistic effect of GC, TNFα and IFNγ during the phosphorylation of STAT3 in MSCs. In view of the multiple steps involved in STAT3 phosphorylation, it is possible that the synergistic action of GC, TNFα and IFNγ may occur in a complex chain. Further studies may delineate the different scenarios.

Glucocorticoid-activating enzyme 11β-hydroxysteroid dehydrogenase type 1 (HSD11B1) converts inactive cortisone to active cortisol and thereby regulates tissue GC levels. We previously reported that IFNγ and TNFα could synergistically stimulate HSD11B1 expression in MSCs [45]. Importantly, HSD11B1 was required for the upregulation of TSG6 by IFNγ and TNFα. Therefore, there exists a mutual reinforcement between GC metabolism and cytokines in augmenting the immunomodulatory function of MSCs.

In summary, in this study, we discovered a novel role of GC in regulating TSG6 expression and thus promoting TSG6-mediated immunosuppression by human MSCs. The results revealed that, in addition to regulating the immune properties of immune cells directly, GC could also act on stromal cells to elicit an anti-inflammatory effect under inflammatory condition. Mechanistically, GC, TNFα and IFNγ function synergistically to active NF-κB and STAT3 to mediate the upregulation of TSG6. The findings reveal that GC could maintain immune homeostasis by acting on stromal cells during tissue injury and further suggest that pretreatment with GC may be considered as a strategy for augmenting the immunomodulatory property of MSCs in clinical settings.

## Materials and methods

### Isolation, expansion, and cultivation of MSCs

Human umbilical cord-derived MSCs were isolated as previously described [11]. MSCs were maintained in low-glucose DMEM (EallBio, Suzhou, China), supplemented with FBS (10%) (Gibco, Massachusetts, USA), penicillin/streptomycin (10 units) (Thermo Fisher, Massachusetts, USA), 10 ng/ml human-bFGF (R&D Systems, MN, USA) and incubated at 37 °C in the presence of 5% CO2. The medium was replaced for every 72 h and the cells were split twice a week. All cells were regularly tested to ensure they were mycoplasma-free. Cells were used before the 12th passage.

### Treatments of MSCs

MSCs were cultured in 12-well or 48-well plates, upon reaching 80–90% confluency, cells were washed with PBS and then treated with indicated stimulations. The stimulations were TNFα (eBioscience, MA, USA) (10 ng/ml), IFNγ (eBioscience, MA, USA) (10 ng/ml), dexamethasone (0.1, 1, 5 or 10 ng/ml) (Sigma, MA, USA), DMSO (Sigma, MA, USA), RU486 (2 μM) (Selleck, WA, USA), BAY 11-7082 (2 μM) (Selleck, WA, USA), nifuroxazide (40 μM) (Selleck, WA, USA) alone or a combination of these two cytokines and inhibitors for indicated times. (*n* = 3 or 4 in each group)

### Real-time PCR

Total RNA was extracted from cell lysate according to the manufacturer’s instructions using FastPure® Cell/Tissue Total RNA Isolation Kit V2 reagent (Vazyme Biotech co.,ltd, Nanjing, China). First-strand cDNA synthesis was performed using PrimeScript™ RT Master Mix (TaKaRa Biotech, Dalian, China) according to the manufacturer’s instructions. The total reaction volume of 10 μL was comprised of 1 ng cDNA, 3 μL DNase/RNase-free water (TaKaRa Biotech, China), 1 μL primers (GENEWIZ, Suzhou, China), 5 μL SYBR qPCR Master Mix (Vazyme Biotech co.,ltd, Nanjing, China). After pipetting the reaction mixture into a 384-well plate, real-time PCR was running by QuantStudio 6 Flex (Applied Biosystems, MA, USA). mRNA levels were calculated referring to β-actin as a housekeeping gene. To compare the expression of each gene caused by different treatment conditions, the fold change of expression was calculated using the equation 2 − ΔΔCt where ΔΔCt = ΔCt (treated) − ΔCt (control), ΔCt (treated) = [Ct (target gene) − Ct (β-actin)], ΔCt (control) = [Ct (target gene) − Ct (β-actin)].

Target genes were normalized to β-actin. Primer sequences are listed in additional file.

### Western blotting analysis

Cells were lysed by RIPA buffer (Beyotime, Shanghai, China) containing PMSF (Beyotime, Shanghai, China) and phosphatase inhibitors (Roche, NJ, USA). Protein samples were separated on a 10% SDS-polyacrylamide gel, and separated proteins were electroblotted onto polyvinylidene difluoride membranes. The membranes were blocked for 1 h with Tris-buffered saline containing 5% albumin bovine serum (BSA) (Amresco, OH, USA) with Tween 20 (TBST) at room temperature with gentle shaking. Then incubated the membranes with primary antibodies GR (12041S, Cell Signaling Technology, MA, USA), Tubulin (2128S, Cell Signaling Technology, MA, USA), p65 (8242S, Cell Signaling Technology, MA, USA), GAPDH (5174S, Cell Signaling Technology, MA, USA), STAT1 (14994S, Cell Signaling Technology, MA, USA), STAT3 (12640S, Cell Signaling Technology, MA, USA), phospho-STAT3 (Tyr705) (9145S, Cell Signaling Technology, MA, USA), for 10 h at 4 °C with gently shaking. The membranes were incubated with HRP-conjugated rabbit secondary antibodies (7074S, Cell Signaling Technology, MA, USA) for 1 h at room temperature with gentle shaking after washing 3 times with TBST for 5 min each. After incubation, membranes were harvested with NcmECL Ultra Kit (NCM biotech, Suzhou, China) according to the manufacturer’s instructions and the signals were detected by Ultra-sensitive automatic imaging analysis system (ProteinSimple, CA, USA). Tubulin or GAPDH was verified and used as an internal control.

### ELISA

TSG6 protein level in the culture supernatant of human UC-MSCs was measured by ELISA as previously described [11]. The supernatant was measured immediately after collection or stored at −20 °C before analysis. Briefly, coated the 96-well plate with TSG6 antibody (sc-65886, clone A38.1.20, Santa Cruz Biotechnology, Texas, USA) at 4 °C overnight, in a coating buffer of 0.2 M sodium bicarbonate (pH 9.2). Washed the plate with PBS, blocked with 0.25% BSA and 0.05% Tween 20 in PBS for 30 min at room temperature, and again washed with PBS. Added samples and a standard of human recombinant TSG6 protein (2104-TS-050, R&D Systems, MN, USA) and incubated for 2 h at room temperature. Then, washed the plate, added biotinylated anti-human TSG6 antibody (BAF2104, R&D Systems, MN, USA) and incubated for 2 h at room temperature. After washing with PBS, added streptavidin-HRP (DY998, R&D Systems, MN, USA) and incubated for 30 min at room temperature and developed using TMB substrate (P0209, Beyotime, Suzhou, China).

### Transfections with siRNA

MSCs were treated with 1 μL INTERFERin® reagent (PolyPlus-transfection, Illkirch, France) along with 1 μL GR, p65, STAT1 or STAT3-siRNA (GenePharma, Shanghai, China). The NC-siRNA was used as control. Washing MSCs with PBS after 48 h and treating MSCs with indicated stimulations. The efficiency of transection was monitored by Western blotting analysis.

### Immunofluorescence staining

MSCs were cultivated in 48-well plates by the stimuli for 1 h, fixed with 4% paraformaldehyde for 20 min at room temperature. Upon washing with PBS three times, the cells were treated for 1 h with a permeabilization and block solution containing 1% Triton X-100 and 5% bovine serum albumin (Sigma-Aldrich, Germany) in PBS. The primary antibody specific for phospho-STAT3 (Tyr705) (9145S, Cell Signaling Technology, MA, USA) was added into the 48-well plate and incubated for 12 h at 4 °C. The samples were further incubated with Goat Anti-Rabbit IgG H&L conjugated with Alexa Fluor® 488 (ab150077, Abcam, Cambridge, United Kingdom) for 1 h at room temperature. After washing, the cells were stained with Hoechst 33342 (C1022, Beyotime, Suzhou, China) for 13 min at room temperature. A high-resolution confocal microscope (TCS SP8, Leica, Wetzlar, Germany) was used to analyze the samples.

### ALI mouse model

Male C57BL/6 mice, 8–10 weeks old, were purchased from Charles River Experimental Animal Technology Co.Ltd. (Beijing, China) and kept under specific pathogen-free conditions. The mice were randomly grouped according to a random number table. Each group included 3, 4 or 5 mice. To induce experimental acute lung injury, 8-week-old C57BL/6 J mice were given 1 mg/kg LPS (L4391-1MG, merck, Darmstadt, Germany) through nasal drip. Human UC-MSCs (2 × 10^5^) pretreated for 24 h with additional stimulations were intravenously injected to treat ALI mice 3 h after the LPS treatment. All experimental mice were sacrificed at 48 h after the LPS treatment. The lungs of mice were collected. The lungs were cut with scissors and digested with 1 mg/mL type one collagenase and 100 μg/mL DNase for 1 h into single-cell suspension. The investigators were blinded to the group allocation when assessing the outcome.

### Flow cytometry

The lung was digested into single-cell suspension, and the red blood cells were removed with red blood cell lysis solution. For the absolute cell number counting, cells were stained by CD45 (103112, biolegend, CA, USA), CD11b (101226, biolegend, CA, USA), Ly6G (127606, biolegend, CA, USA), F4/80 (123114, biolegend, CA, USA), CD80 (104708, biolegend, CA, USA) antibody in the presence of blocking solution (1% BSA in PBS) 30 min at 4 °C and stained with 7-AAD (00-6993-50, eBioscience, MA, USA) 10 min at 4 °C before recording. For the iNOS analysis, cells were stained with Zombie Aqua™ Fixable Viability Kit (423101, biolegend, CA, USA) and stained with CD45 (103116, biolegend, CA, USA), CD11b (101206, biolegend, CA, USA), Ly6G (127639, biolegend, CA, USA), F4/80 (123114, biolegend, CA, USA). And then the cells were fixed with a fixation/permeabilization kit (Invitrogen, CA, USA) at room temperature for 30 min. After the fixation and permeabilization, cells were stained with iNOS (12-5920-82, eBioscience, MA, USA) for 30 min at 4 °C. After PBS washing, cells were recorded using a CytoFLEX (BECKMAN COULTER, Inc.) and analyzed with FlowJo Version 10.1 software (TreeStar, Ashland, OR).

### Statistical analysis

Data was analyzed with Graphpad Prism software (version 8). Data values are presented as means ± SEM of at least three replicates. Each result was confirmed by at least three repeated experiments. Statistical significance was analyzed using two-tailed unpaired student’s *t*-test, one-way analysis of variance (ANOVA) or two-way ANOVA calculation, *P* values < 0.05 were considered statistically significant. *P < 0.05, **P < 0.01, ***P < 0.001,****P < 0.0001.

### Supplementary information

Figure S1

Additional material

WB original data

Reproducibility checklist

## Data Availability

All datasets generated and analyzed during this study are available from the corresponding authors on reasonable request.
