# Supplementary material for: Glucocorticoid activates STAT3 and NF-κB synergistically with inflammatory cytokines to enhance the anti-inflammatory factor TSG6 expression in mesenchymal stem/stromal cells
Source: Cell Death Dis. 2024 Jan 18;15(1):70. doi: 10.1038/s41419-024-06430-1 (PMC10796730; doi:10.1038/s41419-024-06430-1)
Supplement: Supplementary file 1 — Figure S1 [file 41419_2024_6430_MOESM1_ESM.docx]

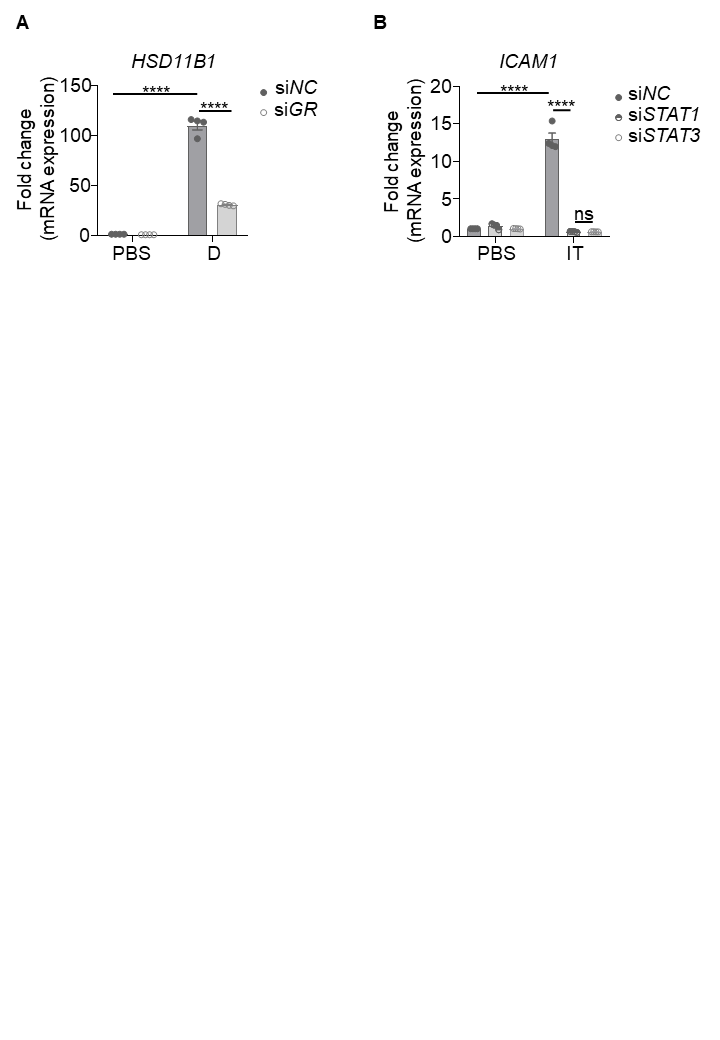


**Figure S1. The transcriptional capacity of GR, STAT1 or STAT3 decreased after they were knocked down.**

**A**. mRNA levels of *HSD11B1* in NC-knockdown MSCs or GR-knockdown MSCs under PBS, D (10 ng/mL) stimulation for 24 h were determined by qRT-PCR. (n=4) **B**. mRNA levels of *ICAM1* in NC-knockdown MSCs or STAT1-knockdown MSCs or STAT3-knockdown MSCs under PBS, IT (10 ng/mL) stimulation for 24 h were determined by qRT-PCR. (n=4)
