# Supplementary material for: Glucocorticoid activates STAT3 and NF-κB synergistically with inflammatory cytokines to enhance the anti-inflammatory factor TSG6 expression in mesenchymal stem/stromal cells
Source: Cell Death Dis. 2024 Jan 18;15(1):70. doi: 10.1038/s41419-024-06430-1 (PMC10796730; doi:10.1038/s41419-024-06430-1)
Supplement: Supplementary file 2 — Additional material [file 41419_2024_6430_MOESM2_ESM.docx]

Real-time PCR was performed using the following primers:

β-actin-F 5’-TTGCCGACAGGATGCAGAAGGA-3’,

β-actin-R 5’-AGGTGGACAGCGAGGCCAGGAT-3’,

*TSG-6*-F 5’-TTTCTCTTGCTATGGGAAGACAC-3’,

*TSG-6*-R 5’-GAGCTTGTATTTGCCAGACCG-3’.

*HSD11B1*-F 5’-AGCAGGAAAGCTCATGGGAG-3’

*HSD11B1*-R 5’-CCACGTAACTGAGGAAGTTGAC-3’

*ICAM1*-F 5’-ATGCCCAGACATCTGTGTCC-3’

*ICAM1*-R 5’-GGGGTCTCTATGCCCAACAA-3’

siRNA sequences:

NC-siRNA sequences were 5′-UUCUCCGAACGUGUCACGUTT-3’ and

5′-ACGUGACACGUUCGGAGAATT-3’.

GR-siRNA sequences were 5′-GAUGUAAGCUCUCCUCCAUTT-3′and

5’-AUGGAGGAGAGCUUACAUCTT-3’.

p65-siRNA sequences were 5’-CCCUAUCCCUUUACGUCAUTT-3’and

5’- AUGACGUAAAGGGAUAGGGTT-3’.

STAT1-siRNA sequences were 5’-GUGGCAAAGAGUGAUCAGATT-3’ and

5’-UCUGAUCACUCUUUGCCACTT-3’

STAT3-siRNA sequences were 5’-CCACUUUGGUGUUUCAUAATT-3’ and

5’-UUAUGAAACACCAAAGUGGTT-3’
