# Supplementary material for: Glucocorticoid activates STAT3 and NF-κB synergistically with inflammatory cytokines to enhance the anti-inflammatory factor TSG6 expression in mesenchymal stem/stromal cells
Source: Cell Death Dis. 2024 Jan 18;15(1):70. doi: 10.1038/s41419-024-06430-1 (PMC10796730; doi:10.1038/s41419-024-06430-1)

**Original data of WB**

Fig 2B GAPDH

Order: 1(siGR), 2(siGR), 3(sip65), 4(sip65), 5(siNC), 6(siNC), 7(siNC), 8(siGR), 9(siNC), 10(siGR), 11(siGAPDH)


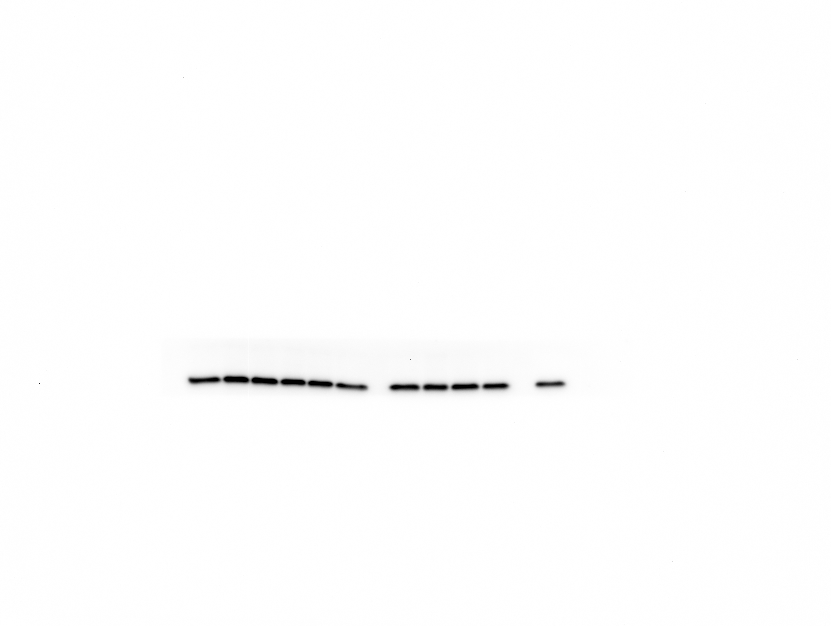


Fig 2B GR

Order: 1(siGR), 2(siGR), 3(sip65), 4(sip65), 5(siNC), 6(siNC), 7(siNC), 8(siGR), 9(siNC), 10(siGR), 11(siGAPDH)


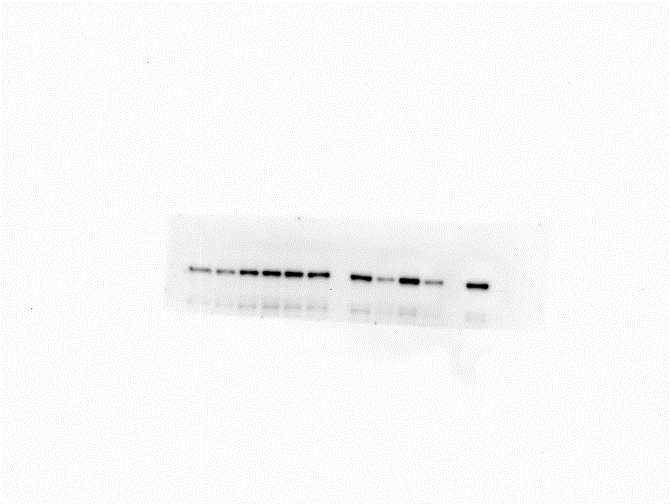


Fig 4B GAPDH

Order: 1(siNC), 2(siNC), 3(siNC), 4(siNC), 5(sip65), 6(sip65), 7(sip65), 8(sip65), 9(sip65), 10(sip65), 11(sip65), 12(sip65)
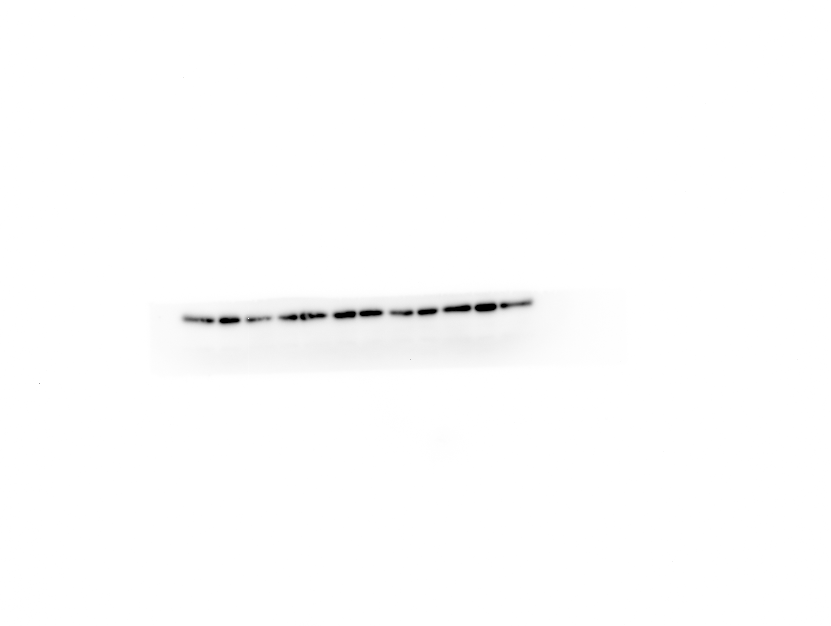


Fig 4B p65

Order: marker, 1(siNC), 2(siNC), 3(siNC), 4(siNC), 5(sip65), 6(sip65), 7(sip65), 8(sip65), 9(sip65), 10(sip65), 11(sip65), 12(sip65), marker, marker


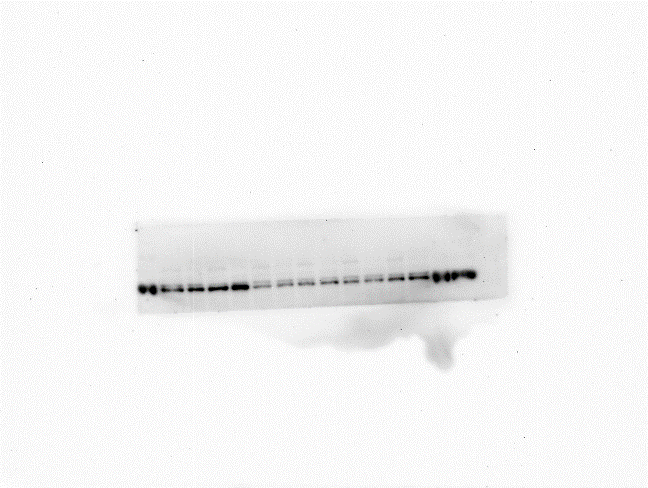


Fig 5B GAPDH

Order: 1(siNC), 2(siNC), 3(siNC), 4(siNC), 5(siSTAT1), 6(siSTAT1), 7(siSTAT1*)*, 8(siSTAT1)


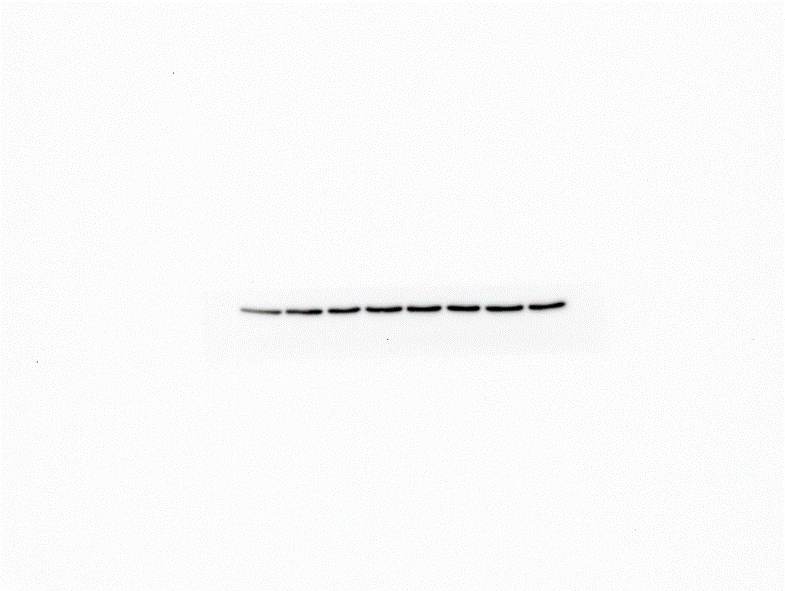


Fig 5B STAT1

Order: 1(siNC), 2(siNC), 3(siNC), 4(siNC), 5(siSTAT1), 6(siSTAT1), 7(siSTAT1*)*, 8(si STAT1)


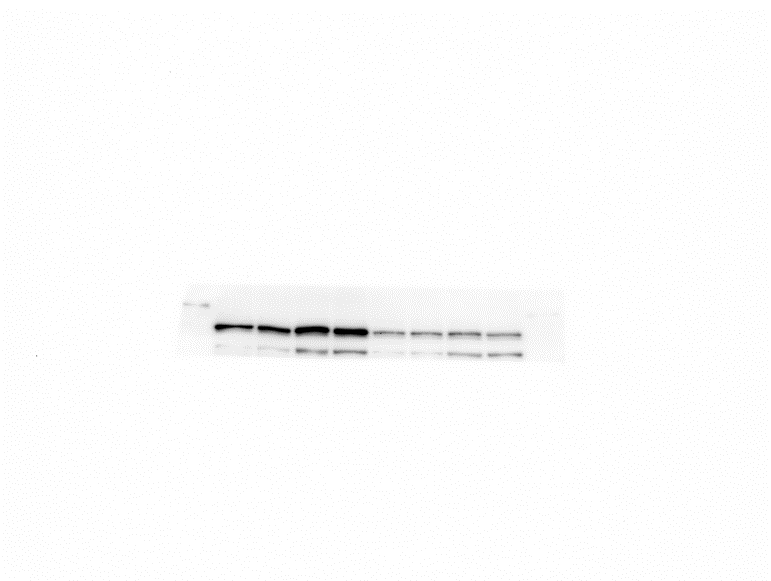


Fig 5D GAPDH

Order: 1(siNC), 2(siNC), 3(siNC), 4(siNC), 5(siSTAT3), 6(siSTAT3), 7(siSTAT3*)*, 8(siSTAT3)


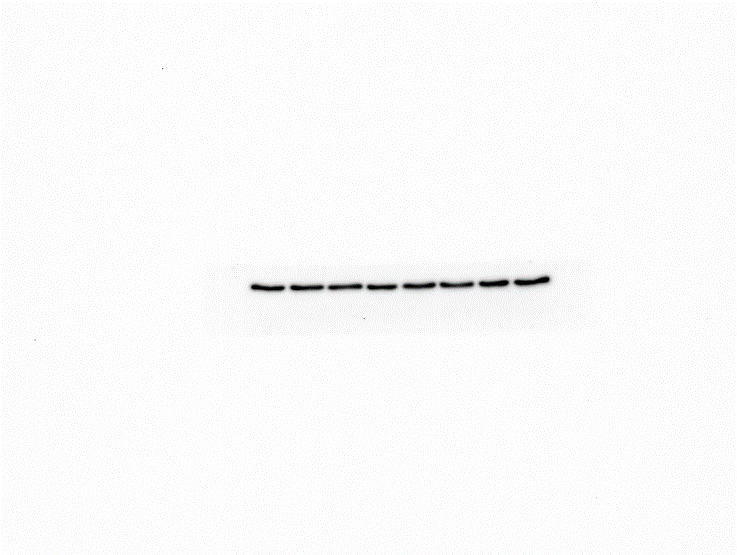


Fig 5D STAT3

Order: 1(siNC), 2(siNC), 3(siNC), 4(siNC), 5(siSTAT3), 6(siSTAT3), 7(siSTAT3*)*, 8(siSTAT3)


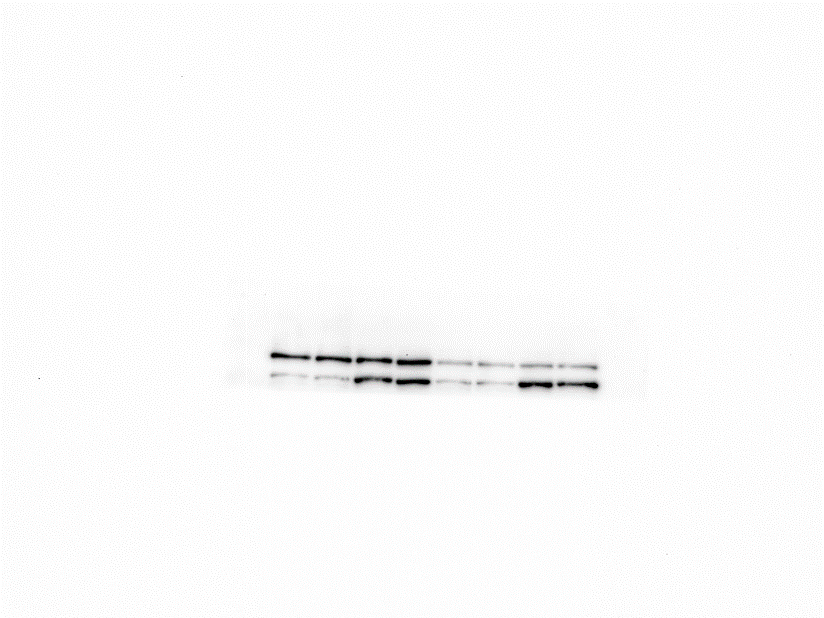


Fig 5G pSTAT3

Order: marker, 1(PBS), 2(D), 3(IT), 4(ITD), 5(D), 6(D), 7(ITD), 8(ITD), marker


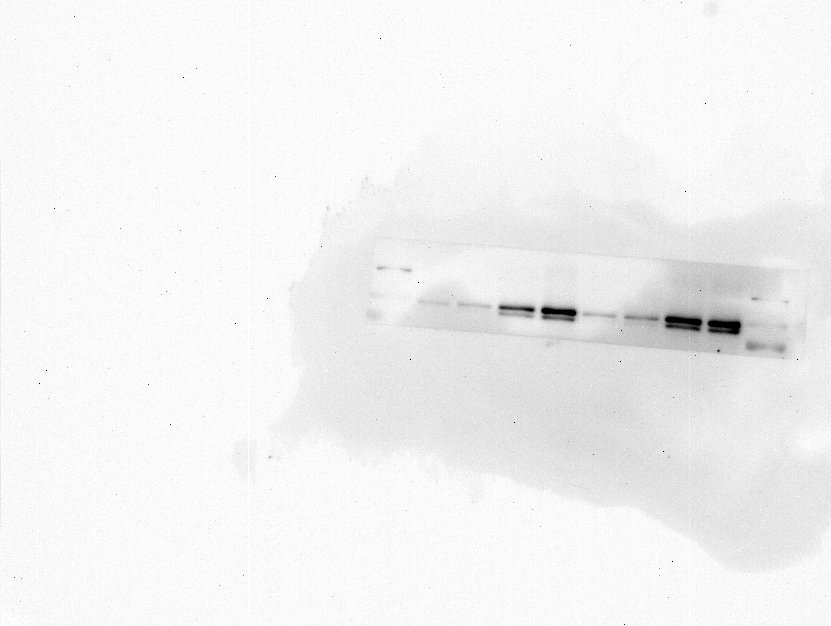


Fig 5G STAT3

Order: 1(PBS), 2(D), 3(IT), 4(ITD), 5(D), 6(D), 7(ITD), 8(ITD)


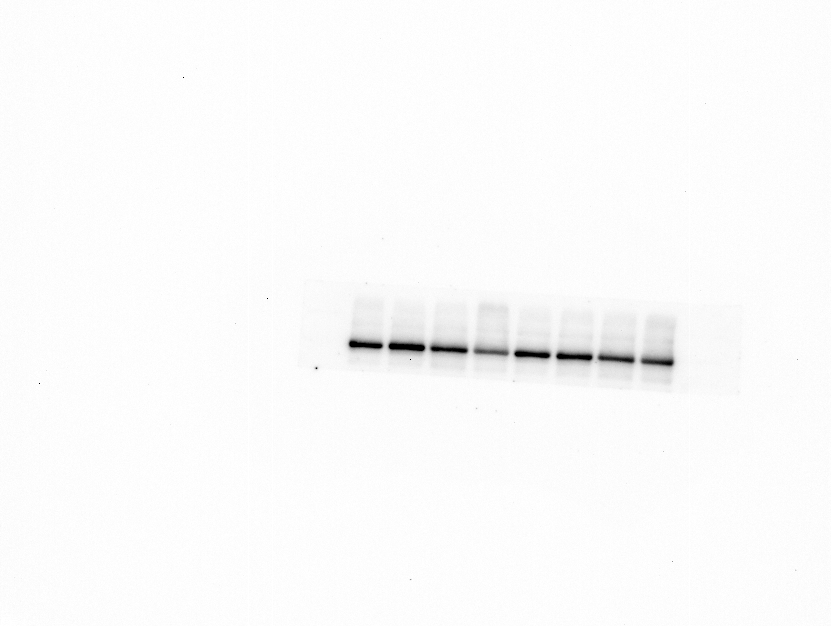


Fig 5G Tubulin

Order: 1(PBS), 2(D), 3(IT), 4(ITD), 5(D), 6(D), 7(ITD), 8(ITD)


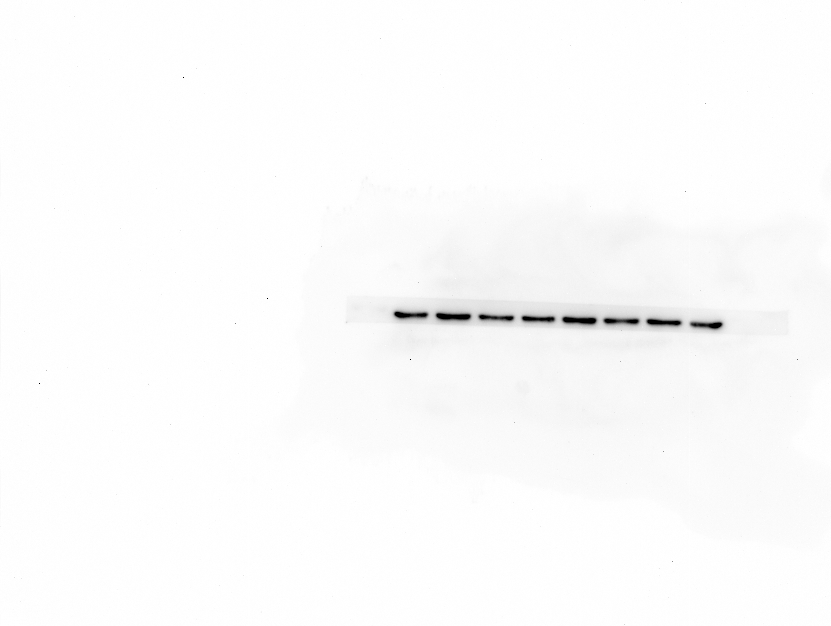


Fig 5H Tubulin

Order: 1(siNC+PBS), 2(siNC+D), 3(siNC+IT), 4(siNC+ITD), 5(sip65+PBS), 6(sip65+D), 7(sip65+IT), 8(sip65+ITD)


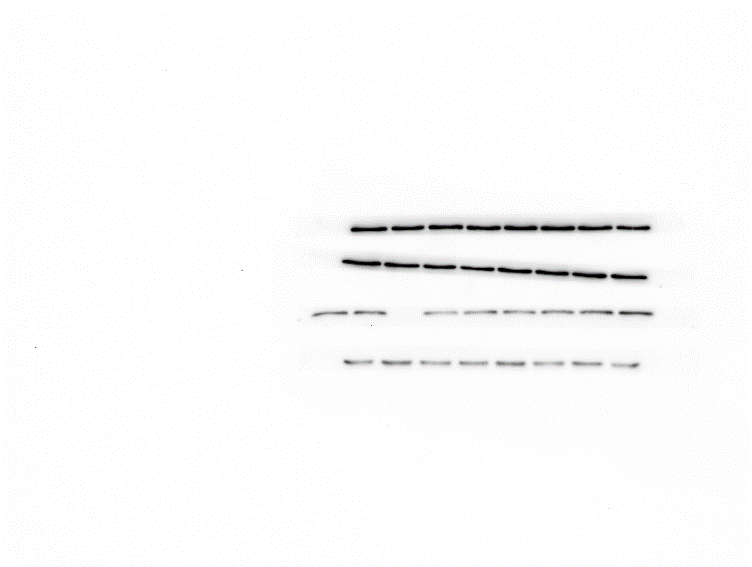


Fig 5H pSTAT3

Order: 1(siNC+PBS), 2(siNC+D), 3(siNC+IT), 4(siNC+ITD), 5(sip65+PBS), 6(sip65+D), 7(sip65+IT), 8(sip65+ITD)


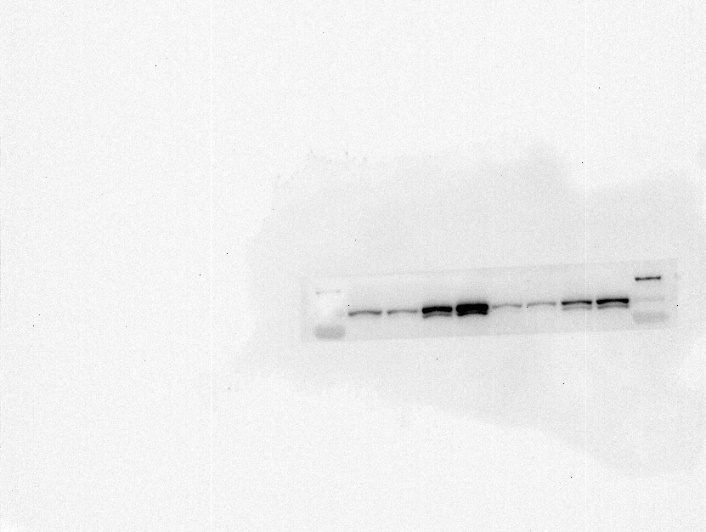


Fig 5H STAT3

Order: 1(siNC+PBS), 2(siNC+D), 3(siNC+IT), 4(siNC+ITD), 5(sip65+PBS), 6(sip65+D), 7(sip65+IT), 8(sip65+ITD)


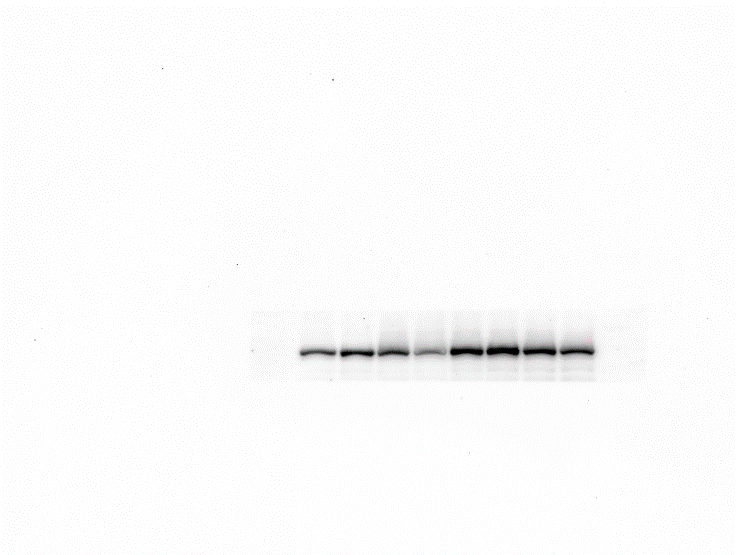

Supplement: Supplementary file 3 — WB original data [file 41419_2024_6430_MOESM3_ESM.docx]
